# Positive health behaviors and their relationships with well-being and work ability of Polish women: Research results

**DOI:** 10.1371/journal.pone.0350232

**Published:** 2026-06-08

**Authors:** Katarzyna Hildt-Ciupińska, Karolina Pawłowska-Cyprysiak, Zofia Mockałło

**Affiliations:** Ergonomics Department, Central Institute for Labour Protection – National Research Institute, Warsaw, Poland; Szkoła Główna Handlowa Warsaw School of Economics, POLAND

## Abstract

**Introduction:**

Lifestyle is considered one of the most important determinants of health, which most of us have an influence on and can modify. However, it is necessary to raise health awareness among people so that they know how to do it effectively.

**Objective:**

This study examines the relationships between health behaviors, well-being, and work ability among Polish female employees.

**Material and methods:**

Anonymous questionnaire surveys conducted among 927 women aged 18−65, using the CAWI method, using a survey developed for the project containing selected standard tools (Work Ability Index, WHO-5 Well-being Index) and a personal survey including personal data and questions regarding, among others: self-assessment of health status and care for it, and well-being.

**Results:**

Women obtained an average result in the Positive Health Behavior Scale; they received an average of 60.3 points out of 102 possible to obtain. Women’s care for their health increased with age; women performing mental work obtained better results than those who performed physical and mixed work. A statistically significant relationship between positive health behaviors and well-being and work ability was demonstrated.

**Conclusions:**

Due to the average care of women for their health, there is a need to constantly raise their health awareness, preferably through health education conducted at the workplace.

## Introduction

According to the World Health Organization, health is “a state of complete physical, mental and social well-being, not merely the absence of disease or disability”. This definition, formulated in 1948, reflects a holistic perspective on health [1]. At that time, however, research on well-being had not yet emerged. It was only several decades later that the field of positive psychology was established, which significantly increased psychology’s focus on well-being as a vital aspect of human health and life [2]. There is no single definition of well-being and various perspectives on this phenomenon exist. In general, well-being is defined as a complex construct that refers to a state of optimal experience and functioning [3]. It integrates mental health and physical health, resulting in a more holistic approach to disease prevention and health promotion [4].

A complex, holistic approach to well-being encompasses a broad range of factors, including mental health, psychological well-being, social and emotional well-being, life satisfaction, economic well-being, development,engaging activities, and work [5].

A more traditional approach, rooted in hedonic philosophy conceptualizes subjective well-being as comprising the presence of positive affect, the absence of negative affect, and the cognitive evaluations of life, such as life satisfaction [6]. A second major strand of well-being research in psychology is the eudaimonic approach, which emphasizes well-being as full functioning [3]. This includes concepts such as psychological well-being [7], defined by dimensions like self-acceptance, positive relations with others, autonomy, environmental mastery, purpose in life, and personal growth, as well as constructs like self-actualization and vitality [8,3].

Well-being is associated with numerous benefits across health, work, family and economic domains. For example, higher levels of well-being are linked to a reduced risk of illness and injury, better immune system functioning, faster recovery, and increased longevity. Individuals with high levels of well-being also tend to be more productive at work [4]. These observed benefits underscore the importance of investigating the multifaceted determinants of well-being across individual and contextual levels.

Well-being is influenced by a variety of factors operating on multiple levels, including health status, social relationships, access to basic resources such as housing and employment [9], individual resources, or the psychosocial environment, both at work and in private life.

One line of research on the predictors of well-being highlights lifestyle as one of the most influential determinants of health [10]. The link between physical health, healthy lifestyle, and well-being has been confirmed in numerous studies [11, 12, 13]. Interventions targeting physical health and lifestyle have also been shown to effectively improve subjective well-being [14, 15, 12]. For example, a recent study demonstrated that lifestyle factors such as not smoking, frequent consumption of fruits and vegetables, and social activity increased the odds of higher psychological well-being over a 10-year follow-up period [16]. Another study based on a large U.S. sample found that salutogenic behaviors (including diet, exercise, and socializing) were associated with higher levels of well-being; however, it also showed that well-being is more easily lost than gained [17]. Nevertheless, the mechanisms underlying the relationship between a healthy lifestyle and subjective well-being remain unclear.

One of the key components of a healthy lifestyle that is strongly associated with well-being is leisure-time physical activity. Recent research indicates that regular physical activity undertaken during leisure time significantly contributes to improved psychological well-being. In a study by Skurvydas et al. [18], leisure-time physical activity was found to be associated with higher levels of happiness, better self-rated health, and a more favorable mood profile. The authors emphasize that recreational and voluntary forms of physical activity promote positive emotions, psychological recovery, and a stronger sense of autonomy, which is a core component of eudaimonic well-being [3]. These findings highlight the importance of considering the quality and context of health-related behaviors, suggesting that leisure-time physical activity constitutes an important resource supporting health and overall well-being.

Lifestyle is crucial for a person’s overall health and the risk of many diseases. Many lifestyle elements, such as diet, smoking, drinking alcohol or lack of physical activity, are potential health risk factors that can significantly affect the incidence and course of diseases, and consequently also life expectancy and the number of years lived in health [19,20], as well as the ability to work. In the context of health, we talk about a health-promoting lifestyle, which Cockerham [21] defined as collective patterns of health-conscious behaviors based on choices from options available to people in accordance with their life chances and are dependent on gender, age, and origin.

Human behavior is a complex phenomenon that includes changes or actions related to external circumstances. When analyzing health behaviors, attention should be paid to many factors that may facilitate the adoption of health-threatening behaviors or disrupt health-promoting behaviors [22].

Each person takes care of their own health differently, which may be due to a different definition of the concept of health care, as well as a different approach to it, sometimes dictated by, for example, health condition. The health behavior of Poles depends on age and other socio-economic and demographic factors. Younger people more often than older people declare regular physical activity and rational nutrition; for older people, on the other hand, using medical services is important. There is no single style of health care among Poles that would combine pro-health behaviors with the use of medical services. Education also differentiates the ways of taking care of health; people with higher education, compared to those with lower education, more often engage in behaviors related to healthy eating and regular physical activity [23].

Lack of sufficient health care may contribute to the development of various ailments and diseases, including those related to work, which may result in a reduced ability to work and, over time, also prevent professional activity; Although the relationship between health care and a healthy lifestyle has not yet been well researched, there is evidence that changing to a healthy lifestyle contributes to improving work ability [24].

Women constitute a specific group of employees due to numerous burdens – both professional work and care responsibilities for dependents (mainly children); unlike men, women are more burdened with these responsibilities [25]. As a result, this may cause (or exacerbate) various health problems and ailments – related to both work and non-work responsibilities, which in turn may result in stress, fatigue, burnout, and physical and mental health problems. Excessive burden of non-work responsibilities may cause a lack of work-life balance, which in turn is associated with poorer health and less care for health. There is a link between high work-life conflict and physical and mental health, low quality of life, high stress [26], as well as low health care, expressed, e.g., by lack of physical activity and undertaking unhealthy behaviors, such as more frequent alcohol consumption [27]. Studies conducted at the Central Institute for Labor Protection – National Research Institute confirmed the link between having a work-life balance – expressed by a positive interaction between work and home – and better health (high self-assessment of health, rarely experienced ailments) and greater health care, compared to people who feel a negative impact of work on home/home on work [28].

The Sustainable Development Goals (Agenda 2030) highlight the significant role of women in sustainable development and emphasize the importance of their leadership in addressing global challenges. Women play many important social roles; they are mothers, caregivers, doctors, teachers, and ministers. One of the 17 Sustainable Development Goals is Good health and well-being (Goal 3), which includes recommendations regarding women’s health. Providing them with equal access to education, health care, economic resources, and work is a fundamental condition for strengthening the role of women in sustainable development. However, only a holistic approach can show the diversity of women and their health that goes beyond their role and reproductive health. An integrated approach to women’s social, biological, and professional roles will allow for the full exploitation of their potential, which is key to sustainable development (Langer et al., 2015).

The present study included women who were economically active at the time of data collection. The decision to focus exclusively on women was driven by both theoretical and methodological considerations. Previous research has consistently shown that women and men differ with respect to health behaviors, psychosocial stressors, mental well-being, and work-related health outcomes. Women are more likely to report higher levels of psychological distress, greater work–family conflict, and different patterns of health-related behaviors compared to men, which may affect their perceived work ability and well-being in distinct ways. Including both genders in a single analytical model could therefore obscure gender-specific associations and limit the interpretability of the findings.

Furthermore, women represent a population that is often underrepresented or insufficiently analyzed in occupational health research, where mixed-gender samples are frequently used without gender-stratified analyses. By focusing solely on women, the present study allows for a more homogeneous sample and reduces variability related to biological, social, and occupational differences between genders, thereby strengthening the internal validity of the analyses.

In addition, women’s working lives are frequently characterized by a cumulative burden of professional and domestic responsibilities, which may uniquely influence their health behaviors, mental well-being, and work ability. Examining these relationships within a female-only sample provides an opportunity to better capture these gender-specific mechanisms and to generate evidence that is directly relevant for designing targeted health promotion and workplace interventions for women.

Thus, the exclusive focus on women is not a limitation but a deliberate research choice aimed at addressing an existing gap in the literature and contributing to a more nuanced understanding of women’s health, well-being, and work ability in the context of paid employment.

### Aim of the study

The aim of the study is to assess health behaviors and determine their relationships with well-being and work ability among women.

## Materials and methods

A cross-sectional questionnaire study was conducted using the CAWI (Computer-Assisted Web Interview) technique.

As part of the study, 927 questionnaire interviews were conducted among women, using a survey booklet developed for this purpose, which included:

- An original survey, developed for the study, containing a metric and questions regarding, among others: self-assessment of health and care for it, self-assessment of well-being. The questionnaire developed for this study was tested in a pilot study among n = 42 women. The questionnaire was clear and understandable for women.
- The Positive Health Behavior Scale for Women [29] – a set of 34 statements grouped into 5 subscales: nutrition, care for the body, safety behaviors, mental health, physical activity. The scale is used to assess individual health care; the results are given in the form of points from 0 to 102; reliability of the entire scale was 0.889. For the subscales: nutrition, physical activity, mental health, body care, Cronbach’s alpha ranged from 0.645 to 0.952;
- Work Ability Index (Tuomi et al. [30]; in the Polish adaptation by J. Pokorski [31]) – a questionnaire for subjective assessment of work ability. It is a tool for estimating the degree to which an employee is able to perform their job (mental and physical abilities). The index includes seven features, each of which is assessed using one or more questions;
- WHO-5 (World Health Organisation – Five Well-Being Index; 1998 [32,33]) – a self-reported, five item measure of mental well-being, in which respondents are asked to assess their wellbeing over the past two weeks by selecting appropriate value on a scale from 0 to 5, where 0 = “At no time”, and 5 = “All of the time”.

### Sampling

For the questionnaire survey, women were selected purposively, based on age (18–65) and type of work performed (mental, physical, mental-physical). In the sample, age was distributed proportionally within the specified age categories; women aged 18–33 constituted 35% of the total, people aged 34–48 constituted 33%, while people aged 49–65 constituted 32%. The type of work was also distributed proportionally in the sample of women surveyed; 34% of women was white collar, 33% blue collar, and 33% mixed.

### Survey organization

Informed consent was obtained from the women for their participation in the study, which consisted of completing an online questionnaire. The consent was obtained by clicking on the informed consent statement. The research was carried out between June and July 2024. The recruitment period for this study was 2–7.06.2024.

The size of the companies in which the surveyed women were employed was distributed roughly proportionally. Nearly 30% of women were employed in large enterprises, 24% in medium-sized and small enterprises, and 21% in microenterprises. Three-quarters of the respondents worked in the private sector, the remainder in the public sector.

The type of work was distributed proportionally among the sample of women surveyed. 34% of white-collar women, 33% blue collar, and 33% performed both white-collar and mixed.

### Ethics committee

The study was approved by the Bioethical Committee at the Institut of Rural Medicine in Lublin (resolution No. 15/2023 of June 20, 2023).

### Statistical analysis of results

The statistical package IBM SPSS Statistics 29 was used for statistical analyses. Basic descriptive statistics were calculated. The significance level was assumed at α = 0.05, and the tests performed were two-sided. Pearson’s chi-square independence test was performed to check whether there was a relationship between the dependent variable and the measured feature. Cramer’s V was calculated to estimate the strength of these relationship. To determine how the groups differed from each other, a test of two proportions with independent groups with the Benjamini-Hochberg correction was performed. In the case of testing differences in variables, a one-way ANOVA analysis of variance was performed for independent groups. Then, Tukey’s HSD post-hoc tests were performed to make multiple pairwise comparisons. In order to determine the significance of differences, in addition to performing Pearson’s r correlation tests, multiple hierarchical regression and a regression model with interaction (moderation) were performed, as well as simple mediation and serial mediation analysis. For this purpose, the SPSS macro PROCESS add-on was used. A multivariate analysis of covariance (MANCOVA) and a single-variate analysis of covariance (ANCOVA) model were used in the study.

## Results

### Characteristics of the respondents

**Fig 1:**
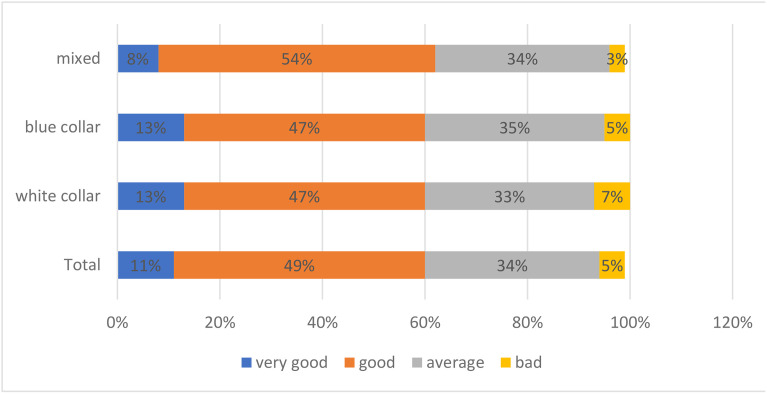
Women’s self-assessment of health, n = 927.

927 women participated in the study. The age distribution in the sample was proportional. Females aged 18–33 constituted 35% of the total sample, females aged 34–48 constituted 33%, while females aged 49–65 constituted 32% of the sample. Considering the remaining demographic characteristics, it can be noted that 23% of the women surveyed lived in villages, 14% in small towns, 27% in medium-sized cities, and 37% in large cities. 33% of the respondents had higher education, 29% secondary, 19% vocational, and 13% post-secondary. 8% had lower education. Nearly 74% of the respondents were in a relationship, and 66% had children. 19% of the women surveyed were caring for a dependent person. Nearly 51% of the respondents assessed their financial situation as average, and 35% as good. The type of work was distributed proportionally in the sample of surveyed women. 34% of women was white collar, 33% was blue collar, and 33% mixed. Only 17% of surveyed women held managerial positions. The size of the companies that employed the surveyed women was distributed more or less proportionally. Nearly 30% of women were employed in large enterprises, 24% in medium and small enterprises, and 21% in microenterprises. Three quarters of the surveyed women worked in the private sector, the rest in the public sector. Self-assessment of women’s health. The survey shows that 60% of women indicated that their health was good (aggregate for the answers “very good” and “good”). 34% gave the answer “so-so”. Only 5% assessed their health as bad. At the same time, no relationship was found between the type of work and general health (χ_((8))^2 = 10.91; p = 0.207), (Fig 1).

### Self-assessment of well-being

Respondents taking part in the study assessed their general level of well-being – in the question “How do you assess your well-being” on a scale of 0 (very low) −10 points (very high) and by completing a 5-question well-being scale, in which respondents could receive 0–25 points (the higher the score, the greater the well-being). The average score obtained in response to the question about self-assessment of well-being was 6.39 points, and on the well-being scale 12.71. There are no significant differences in the declared level of well-being depending on the type of work performed or age (Table 1).

**Table 1:** Women’s well-being, n = 927.

	Well-being –WHO-5 (0–25 points)		Well-being self assessment (0–10 points)	
	M	SD	M	SD
Total	12,71	5,351	6,39	2,07
Type of work				
white collar	12,66	5,37	6,59	2,03
blue collar	12,78	5,69	6,28	2.20
mixed	12,70	4,99	6,28	1,95
Age				
18-33	12,75	5,13	6,38	2,02
34-48	13,10	5,38	6,44	2,11
49-65	12,28	5,55	6.33	2,08

### Work ability

**Fig 2:**
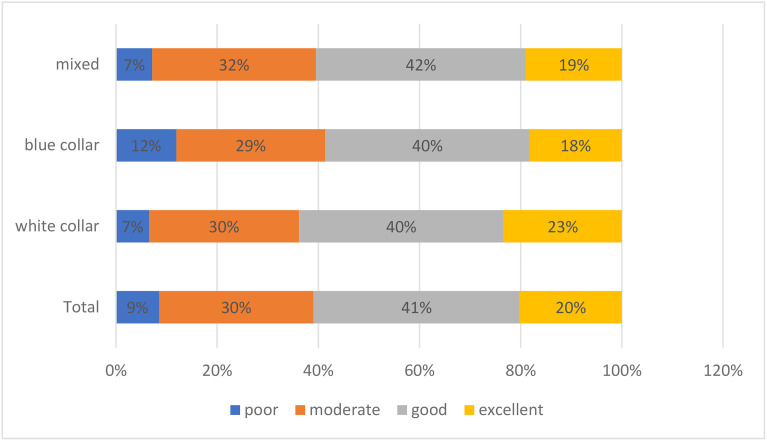
Types of work ability in total and by type of work, n = 927.

The average value of the indicator measured by the Work Ability Index was *M* = 37.48 (*SD* = 6.84); the type of work differentiated work ability – white collar women had a higher work ability than those from the other two groups. Only 20% of women had excellent work ability The most of them had good work ability (Fig 2).

### Positive health behavior scale

The scale includes 34 health behaviors divided into five subscales: nutrition; body care, sleep, rest and mental health; safety; physical activity. The entire scale can be scored from 0 to 102 points. The average number of points obtained by women was 66.4; the minimum score was 21, and the maximum was 92 points.

The average level of pro-health behaviors, measured by the Positive Health Bahaviors Scale scale among the study participants was 60.3 points (0–102 points). Therefore, it can be stated that positive health behaviors are at an average level. A comparison of positive health behaviors was made between groups performing different types of work. The type of work performed significantly differentiates the level of positive health behaviors. In general, white collar women showed a higher level of general positive health behaviors, nutrition, body care, sleep, rest and mental health, and safety behaviors. The type of work does not differentiate pro-health behaviors regarding physical activity (Table 2).

**Table 2:** Positive Health Behaviors Scale, by type of work, n = 927.

Variable	Total		White collar		Blue collar		Mixed		$F_{\left(2;924\right)}$
	*M*	*SD*	*M*	*SD*	*M*	*SD*	*M*	*SD*	
Positive Health Behaviors Scale (0–102 points)	60,30	16,52	63,74	15,26	57,29	17,48	59,75	16,17	12,38***
Nutrition (0–27 points)	14,52	5,64	15,63	5,38	13,56	5,80	14,35	5,57	10,89***
Care for body (0–27 points	16,95	5,59	18,05	5,23	16,10	5,76	16,66	5,61	10,26***
Mental health (0–21 points)	11,22	4,52	11,93	4,39	10,89	4,68	10,82	4,41	5,93**
Safety behaviors (0–15 poits)	11,66	3,04	12,10	2,69	11,08	3,26	11,80	3,06	9,51***
Physical activity (0–12 points)	5,94	2,93	6,03	2,89	5,67	2,95	6,12	2,96	2,04
n	927		315		306		306		

*p < 0,05; **p < 0,01; ***p < 0,001.

The results regarding the Positive Health Behavior Scale and its determinants are described in the article entitled Health behaviors and their determinants in a group of professionally active women, accepted for publication in Occupational Medicine. Workers’ Health and Safety [34].

Statistically significant correlations were noted between age and positive health behaviors, except for the physical activity subscale. Older women scored higher than those from the younger groups (Table 3).

**Table 3:** Positive Health Behaviors Scale, by age, n = 927.

Variable	Age	M	SD	p<
Positive Health Behaviors Scale (0–102 points)	18-33	58,31	17,57	0.00
	34-48	58,31	16,19	
	49-65	64,54	14,81	
	total	60,30	16,52	
Nutrition (0–27 points)	18-33	14,10	5,77	0.00
	34-48	13,95	5,66	
	49-65	15,57	5,35	
	total	14,52	5,64	
Care for body (0–27 points)	18-33	15,96	5,78	0.00
	34-48	16,40	5,55	
	49-65	18,61	5,05	
	total	16,95	5,59	
Mental health (0–21 points)	18-33	11,02	4,69	0.02
	34-48	10.86	4,28	
	49-65	11,80	4,52	
	total	11,22	4,52	
Safety behaviors (0–15 poits)	18-33	11,02	11.12	0.00
	34-48	10,86	11.31	
	49-65	11,80	12,63	
	total	11,66	3,04	
Physical activity (0–12 points)	18-33	6,09	3,02	0.42
	34-48	5,79	2,99	
	49-65	5,92	2,77	
	total	5,94	2,93	

### The relationship between positive health behaviors and well-being and work ability

A statistically significant relationship was found between work ability and behaviors related to sleep, rest and mental health; work ability increases with attention to mental health (Table 4).

**Table 4:** Work ability predictors, n = 927.

Predictor	Work ability	
*B*	***β***
Nutrition	−0,05	−0,04
Care for body	−0,04	−0,03
Mental health	0,24***	0,16***
Safety behaviors	0,12	0,06
Psyhical activity	0,10	0,04

*p < 0,05; **p < 0,01; ***p < 0,001.

Similarly, a statistically significant relationship was found between well-being and behaviours related to sleep, rest and mental health, as well as safety behaviours (at work, at home, in free time). Well-being increased with the care for mental health, and decreased with the lack of safety behaviours (Table 5).

**Table 5:** Well-being predictors, n = 927.

Predictor	Well-being	
	*B*	*β*
Nutrition	−0,02	−0,05
Care for body	−0,00	−0,01
Mental health	0,12***	0,27***
Safety behaviors	−0,07**	−0,11**
Physical activity	0,03	0,04

*p < 0,05; **p < 0,01; ***p < 0,001.

**Fig 3:**
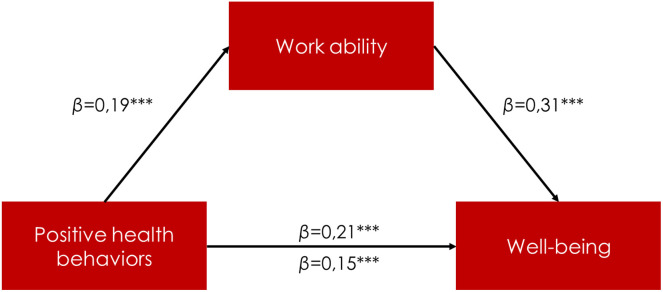
Work ability as a mediator of the relationship between positive health behaviors and well-being.

In both the direct and indirect effect models, positive health behaviors emerged as a significant predictor of well-being (the dependent variable). Work ability was also found to be significantly associated with the dependent variable. The Sobel test indicated a statistically significant mediation effect, suggesting that work ability partially mediates the relationship between health behaviors and well-being. Notably, when work ability was included in the model, the standardized regression coefficient (*β*) for positive health behaviors increased, but remained significant, which supports the presence of partial mediation (Fig 3).

## Discussion

### Main findings

The present study examined the relationships between positive health behaviors, well-being, and work ability among working-age women. The results showed that the overall level of positive health behaviors was moderate, with the highest scores observed in the domain of body care, including preventive health examinations, and the lowest scores in physical activity. Furthermore, positive health behaviors—particularly those related to mental health, sleep, and rest—were significantly associated with both well-being and work ability.

The findings also demonstrated that work ability plays a mediating role in the relationship between positive health behaviors and well-being. Women who reported more favorable health behaviors tended to have better perceived work ability, which in turn was associated with higher levels of well-being.

### Interpretation of the findings

The moderate level of positive health behaviors suggests that although women show some awareness and engagement in health-promoting practices, substantial room for improvement remains, especially in the domain of physical activity. The strong association between mental health–related behaviors and well-being indicates that behaviors with a more immediate impact on emotional and cognitive functioning—such as stress management, sleep quality, and psychological self-care—may be particularly important determinants of subjective well-being.

In contrast, health behaviors related to nutrition, physical activity, and body care were not directly associated with well-being in this study. This may reflect the more distal or long-term effects of these behaviors on mental well-being, compared with the more immediate influence of sleep and mental health care. Physical activity and nutrition may exert their effects indirectly, for example through improvements in sleep quality, body image, or physical fitness, rather than through a direct short-term impact on perceived well-being.

The observed associations between positive health behaviors and work ability suggest that maintaining healthy habits may enhance physical and mental resources necessary to meet work demands. Adequate sleep, rest, and mental health care may improve concentration, emotional regulation, and resilience, while reducing fatigue and sickness absence, thereby supporting sustained work ability.

### Comparison with previous studies

The present findings are consistent with previous research demonstrating that healthy lifestyle behaviors—such as sufficient sleep, regular physical activity, balanced nutrition, and avoidance of harmful substances—are associated with better mental health, mood, emotional regulation, and cognitive functioning [18, 35]. Prior studies have also highlighted the central role of sleep and mental health in psychological well-being and stress regulation [36, 37] (Watling et al., 2016).

The lower levels of physical activity observed among women align with population-level data indicating that women are less physically active than men, often citing lack of time as a major barrier [18] (Gorzelak et al., 2023). Similarly, previous research has shown that blue collar workers tend to exhibit less favorable health behaviors than white-collar workers, a pattern observed in both women and men [ 38,39,40].

The mediating role of work ability identified in this study is in line with earlier findings suggesting that work ability is closely linked to health-related quality of life and well-being [41, 42]. As work ability tends to decline with age, positive health behaviors may act as a protective factor, supporting longer working lives and preventing premature exit from the labor market.

### Practical implications

The findings highlight the need for comprehensive health promotion strategies targeting working-age women, particularly in the workplace. Health education programs should adopt a holistic approach, addressing multiple health behaviors simultaneously, including nutrition, physical activity, sleep, stress management, and mental health care. Special attention should be given to increasing physical activity levels and promoting mental health–related behaviors, which appear to be particularly relevant for well-being and work ability.

Workplace-based interventions may be especially effective, as they offer opportunities to reach economically active women and to address work-related risk factors such as sedentary behavior, high cognitive demands, and psychosocial stress. Supporting women’s work ability through health promotion may not only enhance well-being but also contribute to sustainable employment and the prevention of early retirement.

### Limitations of the study

Several limitations of this study should be acknowledged. First, the cross-sectional design does not allow for causal inferences regarding the relationships between health behaviors, work ability, and well-being. Second, the data were based on self-reported measures, which may be subject to reporting bias. Third, the study sample consisted exclusively of working-age women (18–60 years), which limits the generalizability of the findings to men and other demographic groups. Moreover, the sampling approach may have introduced selection bias, as participation was voluntary and not based on random sampling. Therefore, the study group may not be fully representative of the general population of working women, which should be considered when interpreting the results.

Additionally, potential moderating or mediating variables—such as social support, body image, motivation, or occupational characteristics—were not included in the analyses and should be considered in future research. Longitudinal studies incorporating objective measures of health behaviors and work ability would provide a more comprehensive understanding of the mechanisms linking lifestyle factors, work ability, and well-being.
